# Dentine

**Published:** 1892-03

**Authors:** 


ARTICLE IV.

### DENTINE.

BY MR. MUMMERY.

Before proceeding to the special subject of Dentine he desired to remind them of the views of bone structure and development at present held. The investigations of Professor Sharpey showed clearly that bone is made up of lamellæ, consisting of transparent fibres "decussating with each other in the form of an exceedingly fine network." These transparent fibres rendered visible after decalcification of the bone with acids were according to the recent researches of Professor V. Ebner; made up of finer fibres corresponding in character with bundles of white connective tissue fibres. Treating first of intramembranous ossification as exemplified in the bones of the cranium, if the parietal bone were examined when ossification had already commenced it would be found a mass of bone in the centre, and spicules radiating from it in all directions. These bony radiating spicules would be seen under a microscope to be continuous with bundles of fibres, the osteogenic fibres, extending beyond the circumference of the formed bone. These osteogenic fibres were identical in chemical composition with white connective tissue fibres, and had been described by Gegenbauer as in some situations directly continuous with connective tissue fibres. They were covered with large granular corpuscles, which also filled up the interstices between them. In the last edition of Quain's Anatomy, Professor Schafer had said: "The earthy deposit occasionally appears in an isolated patch here and there on some of the osteogenic fibres in advance of the main area of ossification; Professor V. Ebner considers that the fibres composing these prolongations are not themselves calcified, the uniting substance only being impregnated with lime salts. It would be seen then that in this form of bone development, bundles of fibres, clothed with cells, project from the surface of the already completely calcified tissue. On the long bones, where the chief addition to their substance takes place, beneath the attached periosteum on the outer surface of the bone an arrangement of tissue elements in many respects similar are seen. Applied to the surface of the bone is a very distinct layer of large granular corpuscles, the osteoblasts. These cells are in direct contact with the bone, and occupy the intervals between bundles of fibres of the periosteum which pass deeply into the osseous substance. The arrangement of the fibres in bundles is here very marked, and also their continuity with the connective tissue fibres forming the periosteal membrane. In the peridental membrane similar appearances are met with bundles of fibres from the pericementum enter the cement and persist as the penetrating fibres of Sharpey in that tissue, and occupying the intervals between the bundles we find cells, the osteoblasts and cementoblasts. Some consider that the osteoblasts are directly converted into bone, some cells escaping calcification, as the lacunal cells which become surrounded by the consolidated osteoblasts forming the matrix of the bone. According to other authorities the osteoblasts elaborate the osteogenic substance, not being themselves converted into bone. If it is the case that the protoplasm of the cell is directly converted into bone, as pointed out by Prof. Schafer and others, the outline of the cell areas in the completed tissue ought to be seen, but this cannot be demonstrated, nor can an osteoblast be seen partly converted into bony substance, but if the conversion be correct, surely such cells should be met with.

Directing attention now to dentine, it would be found that it has been set apart among the tissues of the body as a peculiar substance not formed on the plan of other tissues, but standing alone in its mode of development. Two different theories, as in the case of bone, have been held by leading histologists, which may also be described as the conversion and the secretion theories. According to the former, that familiar to students of Mr. Tomes' "Dental Anatomy," and held by Prof. Waldeyer and others in Germany, the odontoblast cells are converted into the matrix of the dentine, and the sheaths of Neumann by direct calcification of their substance, processes from these cells remaining uncalcified as the dentinal fibril. According to the contrary view, that of secretion, the odontoblasts secrete a substance, which, by calcification, becomes the matrix. This view, with modification, is held by Prof. Kolliker, Baume, and others. If the odontoblast cell is converted into dentine by actual alteration of its substance, this must take place in three different degrees, very little, if any, change taking place in the fibril, a peculiar condition being brought about in the part of the cell which forms the sheath of Neumann, rendering it peculiarly resistant to the action of acids, and the rest of the cell becoming converted into the hard substance of the matrix.

As the portion towards the already formed dentine is used up the cell maintains its integrity from beneath, so that from the commencement of calcification to its completion, one cell only is engaged in forming the whole depth of the dentinal substance in and around one tubule. A slow diminution of the calibre of the tubule by a gradual process of calcification of the periphery of the fibril is considered to constantly take place during the life of the tooth.

In a contribution to a discussion in "Nature" on Weissmann's theory of heredity, Professor Cleland made use of this view of the persistency of the odontoblast cell as an illustration.

Mr. Mummery could not but think that the odontoblast is a rather hardly used cell; it has not only had to persist during the whole active life of the tooth, but has had to form the soft fibril, the investing tubule, and the matrix of dentine. The additional function of a nerve ending cell to conduct sensation he thought was hardly consistent with its other offices, and with what was known of the action of cells in other parts of the body. This anomaly struck Mr. Hopewell Smith, in a paper on development, and he would rather consider the function of the odontoblast to be wholly sensory, the cell really being a nerve-end organ and compared by him to the ganglion cells of the spinal cord, other cells than the odontoblasts being concerned in the formation of dentine. In this case, however, the President said, one would expect to find very considerable nerve filaments entering the odontoblasts, and it is also remarkable that in old teeth the odontoblast layer appeared to be absent. Professor Kolliker considered the growth of dentine to be partly by conversion and partly by secretion, that while the central part of the cell persists as the fibril and tubule, the matrix is a secretion from the odontoblasts and other cells of the pulps. Baume holds that the calcification of the dentine takes place in a material secreted by the odontoblasts.

Concerning the relations of the fibril to the pulp, much further investigations was still called for. Professor Klein described the dentinal fibril as arising not from the odontoblast at all, but from a separate layer of deeper cells. With the more perfect means now at our disposal for the examination of dentine in its natural relations to the pulp, it was still very difficult to decide as to the origin of the fibril in mature teeth. Referring to the development of dentine Mr. Mummery said, in examining a longitudinal section of a bicuspid tooth cut by Weil's process and unstained, he saw projecting into the pulp cavity all round the margins, processes like spiculæ. The pulp, though, partially retained, had been torn away in many places from its connection with the dentine. These bundles of fibres—for such they distinctly appeared to be—were evidently continuous with the calcified substance of the dentine on one side and the pulp on the other. The cell layer had fallen away. This specimen seemed so remarkable that he cut and examined many more, and although that was peculiar in the degree of apparent stiffening of the bundles he found that fibres from the pulp passing into the dentine and incorporated with it were very constantly met with.

It stained preparations, where the continuous layer of the odontoblasts was not visible, bundles of fibres were distinctly seen, and in many instances these were crowded with small cells, the nuclei of the odontoblasts being visible in the interspaces between the bundles. This appearance put one very strongly in mind of the osteogenic fibres in membrane bone calcification. If young teeth with incompleted roots were examined, the fine connective tissue fibres were seen passing into the uncalcified portion of the matrix, which was in such teeth proportionally very broad, and the odontoblast cells were seen at the same time forming a very distinct layer, and with separations between them.

Another point shown by these specimens was that a fine striation was visible in the more recently calcified dentine.

Turning his attention to the dentine of other mammalia, the President found in the pulp of the elephant's tusk a very strongly marked connective tissue incorporated with the dentine; and in the incisior tooth of the rat this passage of the connective tissue of the pulp into the dentine was more marked than in any teeth he had examined. Not only could one trace the oblique passage of the fibres into the calcified substance, but in the formed dentine a strongly marked striation parallel with these fibres was very distinct. At the suggestion of Mr. Tomes, Mr. Mummery now turned his attention to fish, and chose as a typical vaso-dentine the teeth of the hake (Merlucius). The strongest confirmation of the conclusion that connective tissue bundles are directly concerned in the process of dentine formation was given by vaso-dentine. He here found a distinct layer of connective tissue fibres or bundles of fibres surrounding the pulp cavity and apparently continuous with the dentine, this layer having been hitherto described as an odontoblast layer by Mr. Tomes. In connection especially with the subject of vaso-dentine, Mr. Mummery desired to acknowledge his great indebtedness to Mr. Charles Tomes.

When these fibres were distinctly visible as a layer, it was very difficult to detect any cells in connection with the dentine, but in other specimens, where the layer was not so marked, rounded cells were seen forming a distinct layer in contact with the dentine, and these cells could be traced into the bone of attachment at the base of the tooth where cells precisely similar in appearance were in contact with the bone.

In many of Mr. Tomes' specimens the substance of the vaso-dentine was seen to be split up into fine fibres similiar in appearance to those surrounding the pulp and running in the same direction at right angles, curiously, to the very obvious longitudinal striation of vaso-dentine. Mr. Tomes had, in recent investigations, also found an outer layer of the dentine apparently made up of decussating fibres.

Mr. Mummery thought that the evidences before them led to the conclusion that dentine like bone was formed of connective tissue. After referring to the strong evidences of the laminar character of dentine, the President said that if the observations of Professor V. Ebner and his own were corroborated and accepted, dentine would be brought out of its isolated position and classed with the connective tissues.—*Dental Record.*

